# “Make America Healthy Again” will make the world sicker: The public health policies of the US administration will have dire results for national and global health

**DOI:** 10.1038/s44319-025-00639-7

**Published:** 2025-11-17

**Authors:** David Robert Grimes

**Affiliations:** TCD Biostatistics Unit, Discipline of Public Health and Primary Care, School of Medicine, Trinity College Dublin, Dublin, Ireland

**Keywords:** Economics, Law & Politics, History & Philosophy of Science, Pharmacology & Drug Discovery

## Abstract

The “Make America Healthy Again” (MAHA) movement are ascendent in the USA, led by Secretary for Health Robert F Kennedy Jr. But despite a superficially laudable intent, MAHA thrives on science denialism, with terrible consequences for public health and trust in medical science.

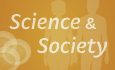

Since Robert F Kennedy Jr took office as US Secretary of Health and Human Services, the Make America Healthy Again (MAHA) movement have been ascendent, implementing a plethora of actions that have caused consternation among public-health officials worldwide. The superficially laudable slogan is chiefly attributed to Kennedy, an environmental lawyer infamous for promoting unevidenced medical claims and his long endorsement of medical conspiracy theories. But MAHA itself is a much broader church, and since Kennedy’s elevation under US President Donald Trump, US health policy has been coopted by MAHA stalwarts. Scientists deemed to be in opposition to Kennedy’s reign have been unceremoniously dismissed, replaced with once-fringe MAHA evangelists, in a process that has so far seen the slashing of more than 20,000 jobs in the NIH, FDA and CDC.

This has been accompanied by a frenzied hunt to find the ostensible causes of America’s woes, far outstripping the cautious pace of medical science. Kennedy’s announcement in April 2025 that the cause of autism would be discovered by September, is an exemplar of precisely this. Beyond the ludicrous timeframe of a mere 5 months for this ambitious undertaking, such a mission statement betrays an intrinsic presupposition that a condition as multi-factorial as autism is caused by some singular, external agent. This reductive conception of Autism-Spectrum-Disorder betrayed a complete misunderstanding of the nuances of epidemiology and causality, prompting criticism from scientists around the world. Kennedy’s appointment of a doctor with highly fringe views who was debarred for foisting expensive and pseudoscientific autism cures on vulnerable patients only further underscored the impression that this was less scientific investigation, more pseudoscientific show trial. The culmination was the administration’s high-profile announcement in September that acetaminophen use during pregnancy was the cause of autism. This assertion has been roundly condemned by scientists as a spurious correlation, given that it fails to manifest in larger studies and lacks any known mechanistic validity.

Acetaminophen is the latest volley in the administration’s assault on scientific consensus, which has also seen the banning of certain artificial food dyes and sweeteners, despite a lack of credible evidence to justify such actions, and a disregard for the unnecessary public concern generated. At Kennedy’s behest, the CDC have stopped recommending water fluoridation, leading to states like Utah passing legislation banning water fluoridation despite its well-established public-health benefits.

But while all these measures are contrary to best evidence and undermine public trust in science, it is vaccination that is the most concerning target of both Kennedy and the administration. In a fundamental undoing of decades of established policy, Kennedy ousted all independent vaccine advice from the Advisory Committee on Immunization Practices. In their stead, he has appointed a menagerie of closely allied contrarians, many of whom had long histories of antivaccine activism. Alongside this has been the unilateral ending of funding for mRNA-vaccine research, halting vital insights not only into immunisation but also cancer treatment where these approaches have untold utility.

… it is vaccination that is the most concerning target of both Kennedy and the administration.

## IDEOLOGICAL MOTIVATIONS

To understand these actions, it is crucial to understand the ethos and ideological motivations underpinning MAHA. The movement itself attracts a diverse coalition, from “health freedom” campaigners who perceive public-health measures as impositions on personal liberty, to health-conscious parents concerned about their children’s well-being, to alternative health practitioners who advocate for “natural” or “integrative” cures in lieu of conventional medicine, to high-profile “wellness” influencers and social-media figures. These motivations are diverse and underlying beliefs often mutually contradictory, but are united in a reductive view of health, a distrust of conventional medicine and an implicit rejection of the socio-economic determinants of health (Box 1). A darker undercurrent also persistently breaks through the surface: the deep intersection of MAHA beliefs with conspiracy theories about medicine.

A darker undercurrent also persistently breaks through the surface: the deep intersection of MAHA beliefs with conspiracy theories about medicine.

This manifests most obviously in the form of anti-vaccine propaganda. Anti-vaccine activists are integral to the MAHA movement, and several prominent figures have acquired positions of influence under Kennedy. This tangible antipathy to vaccines, especially public-health measures more broadly, has seen media outlets from *Le Monde* to the *New York Times* label Kennedy and MAHA compatriots’ vaccine sceptics. This designation is a well-meaning error and too often a gateway to false balance, giving poorly evidenced views the illusion of respectability (Grimes, 2019a). Scepticism is crucial to probing whether a particular hypothesis is supported by evidence, a vital element of the scientific process. Questioning the safety and efficacy of vaccines, for instance, is perfectly legitimate and the very reason vaccine advisory bodies exist. But crucially, scepticism demands arriving at conclusions supported by evidence. When one chooses to ignore or denigrate evidence, rendering their prior conviction untenable, this is the very antithesis of scepticism.

In Kennedy’s case, there is an overwhelming public record that he has for decades not only persisted in ignoring empirical evidence against his claims on health but has been a major spreader of medical disinformation. Prior to the pandemic, his foundation was the world’s leading funder of anti-vaccine propaganda on Facebook (Jamison et al, 2020). At the dawn of COVID-19, Kennedy was one of the “Disinformation dozen”, 12 individuals responsible for vectoring over 65% of pandemic misinformation (Nogara et al, 2022). His embrace of pseudoscience is well-documented (Box 2), and tellingly, he refused to accept correction or criticism, demonstrated by his recent fiery exchange with US senators. These actions are exemplars of motivated reasoning, when one starts with a conclusion and cherry-picks or even bends data to justify a prior position, ignoring evidence to the contrary. This isn’t scepticism, but unadulterated denialism – the very antithesis of critical thought.

Far from being a sceptic, Kennedy has long bent evidence to fit conclusions instead of adapting to the evidence as the scientific method demands. This was laid bare in May, when citations in a much-hyped report by the MAHA commission—taking aim at MAHA’s usual targets of childhood vaccines, ultra-processed foods, pesticides and prescription drugs—were found to not actually exist, despite Kennedy proclaiming it “gold standard” science. Instead, the report appears to have been, in part, at least AI-generated. This exercise in buttressing dubious science also must be seen in context of Kennedy’s slamming of medical journals including *The Lancet*, *JAMA* and the *New England Journal of Medicine* as “corrupt”, threatening to ban federally funded scientists from publishing in them.

Far from being a sceptic, Kennedy has long bent evidence to fit conclusions instead of adapting to the evidence as the scientific method demands.

Box 1 Common tenets of MAHA Beliefs

- America is enduring an “epidemic” of chronic illness, primarily affecting children, and this epidemic is driven by poor dietary choices and exposure to toxins.
- These ostensible toxins are typically nebulous, inconsistent and poorly defined, and asserted without or in contradiction of medical or biological evidence.
- Underlying conviction that the food, chemical and pharmaceutical industry are conspiring to hide the ill-effects of their products on American health.
- Deep opposition to vaccination, fluoridation and other public-health measures, with conspiracy theories about these practices commonly held.
- Invocation of the appeal to nature fallacy, and intrinsic affinity for products and supplements deemed to be ‘natural’, regardless of the merit of this claim.
- Advocating of special diets, unevidenced supplements and various elixirs and technology for optimising health.
- Reductive views of medical science, and insistence that complex conditions like autism or mental-health issues have simple “root causes”.
- A fixation on detoxification is rooted in the idea that most health problems are caused by toxins and thus treated by removing them.
- Frequent adoption of ideas from alternative and complementary medicine and discredited medical theories from antiquity.
- Rejection of evidence-based medicine and medical science as corrupt or unreliable, and reliance on anecdotes over data.
- Downplaying of social determinants of health, systematic factors and health inequality, instead typically advocating for individual actions.

Box 2 Robert F Kennedy’s track record of Health disinformation

- **Vaccination:** Repeatedly claims that vaccines (and specifically thimerosal in vaccines) is dangerous and that vaccines cause neurological damage and autism. Insists vaccines are under-tested and that vaccine injuries are under-reported, stating, “*this is a Holocaust, what this is doing to our country*.” During his tenure, Children’s Health Defense (CHD) became a leading producer of anti-vaccine propaganda, including championing disgraced Andrew Wakefield.
- **Electromagnetic radiation:** Has claimed that 5G and Wi-Fi, as well as other wireless radiation, may open the blood–brain barrier, causing cancer, damaging mitochondria and contributing to autism and chronic disease. Kennedy stated that 5G “..*causes cancer. It causes DNA dysfunction. It penetrates the blood–brain barrier. It’s making our children stupider and sicker.”*
- **Water Fluoridation:** Opposes community water fluoridation, insisting that fluoride reduces IQ, and causes cancers, bone issues and thyroid disease. Believes fluoride is an industrial waste dumped in water, and pushed President Trump to ban it, labelling it a *“dangerous neurotoxin”*.
- **HIV/AIDs:** He has made statements that appear to question whether HIV truly causes AIDS and suggested that evidence around HIV/AIDS has been misrepresented. In 2021, he claimed Anthony Fauci deliberately sabotaged AIDs research, spoke positively of the long-debunked Duesberg hypothesis of AIDs denialism, condemning the *“theology that HIV is the sole cause of AIDS”*.
- **Germ Theory**: Has expressed a preference for long-debunked theories of Louis Pastuer’s rival Antoine Béchamp claiming that disease stemmed from the state of the body, decrying the *“domineering ascendancy of germ theory as the cornerstone of contemporary public-health policy”* further insisting that *“..a $1 trillion pharmaceutical industry pushing patented pills, powders, pricks, potions and poisons and the powerful professions of virology and vaccinology … fortifies the century-old predominance of germ theory.”*
- **Anti-depressants**: Has implied that anti-depressant drugs are linked to school shootings, stating these shootings *“really started happening coterminous with the introduction of these drugs, with Prozac and with other drugs.”*
- **Unproven and alternative therapies:** Tweeted in October 2024 that the “*FDA’s war on public-health is about to end. This includes its aggressive suppression of psychedelics, peptides, stem cells, raw milk, hyperbaric therapies, chelating compounds, ivermectin, hydroxychloroquine, vitamins, clean foods, sunshine, exercise, nutraceuticals and anything else that advances human health and can’t be patented by Pharma. If you work for the FDA and are part of this corrupt system, I have two messages for you: 1. Preserve your records, and 2. Pack your bags”*

## ECHOES OF LYSENKOISM

Distorting evidence to fit ideology has grim historical precedents (Grimes, 2019b). Trofim Lysenko was a plant biologist in the USSR whose interests in agriculture were outstripped by his devotion to Soviet Ideology. Rejecting the very idea of genes and instead insisting that acquired characteristics could be passed on to offspring, Lysenko claimed his ideas would revolutionise food production. Such assertions resonated with party propaganda of ingenious workers solving practical problems to outwit the bourgeois, and Lysenko was lauded and elevated in the hierarchy of the Communist Party. His ringing endorsement by Stalin stood in stark counterpoint to the concerns of Soviet scientists, however, who saw clearly even at the time that the agronomist’s claims were simply wrong.

Lysenko’s lack of scientific training translated into poorly controlled, subpar experiments, and nor was he above supporting his claims with fraudulent data to bolster his heroic image. He was especially appalled by Darwin’s work on competition in natural selection and decried the theory of evolution as inherently anti-communist. As a result, Lysenko held the entire field of genetics in contempt and used his growing political means to bolster his dubious science. Adamantly rejecting standard scientific practices such as control groups and statistical methods, Lysenko insisted only his assertions were true science. But so egregious were his errors that other scientists showed his work to be either unjustified or blatantly falsified. Denouncing biologists as “fly-lovers and people haters”, he insisted they were effectively “wreckers” seeking to subvert and sabotage the USSR.

When Stalin granted him the power to pursue scientific critics, Lysenko in 1948 officially declared genetics ‘fascist’, lambasting it as a ‘bourgeois perversion’. The Politburo outlawed the teaching of genetics across the USSR, and genetic research was forbidden. Geneticists across the USSR were unconditionally fired, their work publicly condemned, and approximately 3,000 scientists were executed or sent to gulags and prisons. Persecuted scientists in genetics, biology and medicine were replaced by incompetent sycophants loyal to Lysenko. But the consequences for ignoring reality are severe. Lysenko’s pseudoscience culminated in hugely reduced crop yields across the USSR, leading to mass starvation. This metastasised across borders to China, where Lysenkoism was a core foundation of Mao’s agricultural policies that failed so disastrously in the 1950s, culminating in the Great Chinese Famine which left between 15-45 million people dead.

## LYSENKOISM IN HEALTH POLICY—AIDS DENIALISM

Lysenko’s stranglehold over Soviet science only ended in 1964, following a coup by Soviet nuclear scientists who finally felt emboldened enough to remove him, but his story goes far beyond the hubris of one man: it is a cautionary tale for what transpires when we not only ignore evidence, but seek to suppress it. And we need not look far to see grim historical analogues in health policy. In 1999, Thabo Mbeki’s South African government embraced AIDs denialism popularised by fringe figures like Peter Duesberg despite soaring national infection rates. Decrying antiretroviral drugs as big-pharma conspiracy, health minister Manto Tshabalala-Msimang denied them for patients, instead advocating alternative medicines and diets to treat HIV/AIDs, leading exasperated medical professionals and South Africans to bestow the barbed title of “Dr Beetroot” upon her (Grimes, 2019b).

In response to the worsening crisis and unchecked ascendancy of pseudoscience, more than 5000 scientists and clinicians signed the Durban declaration, asserting unambiguously that HIV was indeed the cause of AIDs. Rather than countenance any of this, Mbeki’s government insisted on ideology over evidence, elevating the handful of fringe figures that reinforced their stance while dismissing the incontrovertible scientific consensus of the Durban declaration. Such folly had deadly consequence—by 2008 it was estimated that over 330,000 people would die because of South African AIDS denialism (Chigwedere et al, 2008). This did not stop denialists attempting to downplay or even deny outright these estimates—a non-peer-reviewed counter by Duesberg continued to insist there was “no proof that HIV causes AIDS” before its eventual retraction. Current estimates place the death toll at between 343,000 and 354,000 people in South Africa succumbing to avoidable deaths, a direct consequence of the Mbeki administration’s rejection of science (Chigwedere and Essex 2010). Even today, the shadow of AIDS denialism still means that HIV and AIDs are significantly under-reported in South Africa (Groenewald et al, 2025).

## VALID CONCERNS BUT FALSE PROMISES

As concerning as things are, it need not end in tragedy. First, we need to recognise that at least some of the allure of MAHA reflects a genuine crisis and entirely understandable frustration. The USA spends much more on healthcare per capita than any other nation, with poorer outcomes (Tanne, 2024). Nonetheless, Americans have a life expectancy of 78.4 years, versus 84.5 in comparable countries, a difference of 4.1 years. Health inequality is much starker than in European nations, with a complex and opaque system of insurers and obscenely wealthy healthcare providers gatekeeping even basic access to care. Unlike most of the world, pharmaceutical companies market directly to consumers, with cases of physicians being rewarded for pushing drugs on patients, and a culture of over-medicalisation to define or treat normal life issues. The net result is that sick people are often denied healthcare, while others of greater means may be over-treated. Disillusionment with healthcare in the USA is not irrational, but a direct response to high costs, poor outcomes and staggering systemic inequality.

…we need to recognise that at least some of the allure of MAHA reflects a genuine crisis and entirely understandable frustration.

But these legitimate grievances and the ensuing search for alternatives create an opening for both the misguided and charlatans to exploit suffering, capitalising on frustration to profiteer with false, even harmful, alternatives. Instead of recognising the systemic inequalities and structural factors, MAHA instead hunts for “toxin” scapegoats on which to pin blame: vaccines, fluoride, 5G, seed oils. Instead of recognising that the profit-driven nature of US healthcare is a problem, they instead hawk individualistic and ineffective solutions from elixirs to supplements. Any empowerment this might seem to generate is ultimately illusory, misleading the vulnerable and passing responsibility for mending a broken system back onto individuals.

## A GLOBAL THREAT

It might be tempting to dismiss these problems as uniquely American, which the rest of the world can simply choose to ignore. But the reality is that due largely to social media, this is not the case. The conspiracy theories of MAHA are not confined to the USA—they metastasise across the world and crop up in populist movements from Europe to South America. Left unchallenged, this mendacity has a corrosive influence on public health which it disparages so viciously worldwide. The ultimate result is increased harm to everyone, especially the most disadvantaged in society and those with the lowest health literacy.

The conspiracy theories of MAHA are not confined to the USA—they metastasise across the world and crop up in populist movements from Europe to South America.

This has been evident in the worrying rise of vaccine hesitancy, which already had been a huge problem before the pandemic. It has increased in scope not only in the USA but throughout the world, with a dark renaissance in once-conquered diseases (Grimes, 2022a). Exposure to anti-vaccine propaganda is a major factor for vaccine hesitancy (Jolley and Douglas, 2014), and the brunt of the explosion of measles cases in the USA this year hit the youngest hardest, with children dying from an illness declared eradicated in the States a mere 25 years ago (Hotez, 2025). But this is not solely an American problem—record outbreaks of once virtually conquered diseases have rocked Europe too, and even before the pandemic, the WHO were forced to declare vaccine hesitancy a top 10 threat to public health. Health misinformation is endemic on not just vaccines, but on drugs, food and even cancer (Suarez-Lledo & Alvarez-Galvez, 2021; Do Nascimento et al, 2022; Grimes, 2022b; Swire-Thompson and Lazer, 2020), and while these fictions are embraced by MAHA, they are virulent and easily spread far beyond the USA. Even if such effects were just confined to America, the situation would still be grim for global health, as it would at best render America a breeding ground for infectious diseases that would inevitably transcend borders in our interconnected world.

As scientists, our primary obligations are to honest inquiry and human well-being. We simply cannot afford to become fatigued or apathetic at this juncture. It is imperative that we not only collectively challenge asinine and toxic pseudoscience on health but offer a better reality than MAHA’s lurid fictions. Intellectual humility and transparency are critical in this undertaking, as is compassion: it is crucial to not only recognise why people are susceptible to these messages, but to explore how we can do better, together.

Even in this era of disinformation and reduced trust in institutions, physicians and scientists remain the most trusted groups in society. This will also include challenging the small but vocal cohort of physicians and scientists beloved by MAHA, who peddle conspiracy theory and medical misinformation (Grimes and Greenhalgh, 2024; Sule et al, 2023; Saver, 2024). While these individuals constitute a fringe, they undermine public understanding and trust in medical science, and it is critical that regulatory bodies and fellow professionals do not allow them to spread disinformation unopposed.

The fundamental problem is that MAHA offers a superficially alluring premise, but it is one rooted in dubious and harmful fictions, with global ramifications beyond the borders of the USA. Beneath a reasonable veneer, MAHA pivots on pseudoscience and conspiracy theory, with corrosive influence on both public understanding and public trust in science and medicine. By addressing the genuine problems in healthcare that make people susceptible to movements like MAHA, physicians and scientists can offer better alternatives to help ensure a healthier and more informed society.

By addressing the genuine problems in healthcare that make people susceptible to movements like MAHA, physicians and scientists can offer better alternatives to help ensure a healthier and more informed society.

The rise of MAHA is something that scientists and physicians cannot and should not ignore. Only united in promoting evidence, transparency and public understanding can we hope to counter the enduring harms from modern Lysenkos and expose that peddling snake-oil and conspiracy theories does not “Make America Healthy Again”—it makes everyone sicker, more divided and less informed. Only by uniting and unifying our voices to advocate for evidence-based policy, can we offer better alternatives to the insidious disinformation underpinning MAHA and address the real and systemic problems facing not just Americans, but all citizens of the world.

## Supplementary information

Peer Review File
