# Supplementary material for: “Make America Healthy Again” will make the world sicker: The public health policies of the US administration will have dire results for national and global health
Source: EMBO Rep. 2025 Nov 17;26(23):5639–43. doi: 10.1038/s44319-025-00639-7 (PMC12678486; doi:10.1038/s44319-025-00639-7)
Supplement: Supplementary file 1 — Peer Review File [file 44319_2025_639_MOESM1_ESM.pdf]

EMBO reports

"Make America Healthy Again" will make the world sicker

David Robert Grimes

Corresponding author(s): David Robert Grimes (davidrobert.grimes@tcd.ie)

Review Timeline:

Submission Date:	6th Oct 25
Accepted:	31st Oct 25

Editor: Holger Breithaupt

Transaction Report: This article was editorially reviewed.
